# Quantification of Signal-to-Noise Ratio in Cerebral Cortex Recordings Using Flexible MEAs With Co-localized Platinum Black, Carbon Nanotubes, and Gold Electrodes

**DOI:** 10.3389/fnins.2018.00862

**Published:** 2018-11-29

**Authors:** Alex Suarez-Perez, Gemma Gabriel, Beatriz Rebollo, Xavi Illa, Anton Guimerà-Brunet, Javier Hernández-Ferrer, Maria Teresa Martínez, Rosa Villa, Maria V. Sanchez-Vives

**Affiliations:** ^1^Systems Neuroscience, Institut d’Investigacions Biomèdiques August Pi i Sunyer (IDIBAPS), Barcelona, Spain; ^2^Instituto de Microelectrónica de Barcelona, Centro Nacional de Microelectrónica, Consejo Superior de Investigaciones Científicas, Barcelona, Spain; ^3^Centro de Investigación Biomédica en Red en Bioingeniería, Biomateriales y Nanomedicina, Madrid, Spain; ^4^Instituto de Carboquímica, Consejo Superior de Investigaciones Científicas, Zaragoza, Spain; ^5^ICREA, Barcelona, Spain

**Keywords:** SNR, neural recording, slow oscillations, low impedance, neural interfaces

## Abstract

Developing new standardized tools to characterize brain recording devices is critical to evaluate neural probes and for translation to clinical use. The signal-to-noise ratio (SNR) measurement is the gold standard for quantifying the performance of brain recording devices. Given the drawbacks with the SNR measure, our first objective was to devise a new method to calculate the SNR of neural signals to distinguish signal from noise. Our second objective was to apply this new SNR method to evaluate electrodes of three different materials (platinum black, Pt; carbon nanotubes, CNTs; and gold, Au) co-localized in tritrodes to record from the same cortical area using specifically designed multielectrode arrays. Hence, we devised an approach to calculate SNR at different frequencies based on the features of cortical slow oscillations (SO). Since SO consist in the alternation of silent periods (Down states) and active periods (Up states) of neuronal activity, we used these as noise and signal, respectively. The spectral SNR was computed as the power spectral density (PSD) of Up states (signal) divided by the PSD of Down states (noise). We found that Pt and CNTs electrodes have better recording performance than Au electrodes for the explored frequency range (5–1500 Hz). Together with two proposed SNR estimators for the lower and upper frequency limits, these results substantiate our SNR calculation at different frequency bands. Our results provide a new validated SNR measure that provides rich information of the performance of recording devices at different brain activity frequency bands (<1500 Hz).

## Introduction

Interfacing the brain using electrodes to record from and stimulate it is a standard approach for investigating brain function. Multielectrode arrays (MEAs) in particular are widely used devices, both in basic research and in the clinic, that can record electrophysiological signals simultaneously from different neuronal populations. MEAs have been used in basic research to study brain function in *in vitro* experiments (Berdondini et al., 2006; Taketani and Baudry, 2006; D’Andola et al., 2017) as well as in anesthetized and in chronically implanted behaving animals (Nicolelis et al., 2003; Dickey et al., 2009; Khodagholy et al., 2015). Furthermore, the use of chronic implants based on MEA technology has increased in the last few decades with the development of brain-computer interfaces (BCIs) to compensate for lost neural functions (Hochberg et al., 2006; Lebedev and Nicolelis, 2006; Donoghue, 2008). An important group of BCIs is based on the recording of the local neuronal population such as local field potentials (LFPs) and on multi-unit activity (MUA) (Lebedev and Nicolelis, 2006). An important drawback of this group of BCIs is their limitations on obtaining brain signals within a large bandwidth, especially high-frequency signals such as MUA. These limitations are not only a feature of the electrodes themselves, but also a feature of the design of the recording system and the filtering procedure. Hence, one of the strategies to overcome this frequency limitation consists in increasing the ability of the recording system to accurately sense and record biological signals in their whole frequency range in order to detect as many frequency bands as possible. The main feature that characterizes an ideal extracellular microelectrode for recording brain signals is a high signal-to-noise ratio (SNR), which is a measure of the fidelity of the received message for the whole frequency band containing useful neural information (Baranauskas et al., 2011). SNR is usually assessed using saline-measured electrode impedances. Typically, electrodes are made of metallic conductors such as gold (Au), and since the electrodes used in MEAs are on the micrometer scale, it is a challenge to achieve low electrode impedance with plain conductors only (Obien et al., 2015). It is generally assumed that higher SNR values can be achieved by lowering the electrode impedance, which decreases with increasing active surface area. Thus, recent research has pursued new materials and fabrication techniques aimed at increasing as much as possible the active surface area. Two particularly appealing approaches to increasing the active surface area of electrodes are: (1) platinum black coating electroplated on metallic electrodes (Pt) (Desai et al., 2010; Zhang et al., 2013); and (2) polypyrrole/carbon nanotubes composite electrodeposited on metal electrodes (CNTs) (Keefer et al., 2008; Baranauskas et al., 2011). Whereas the quantitative characterization of the working performance of electrodes is usually achieved by using electrochemical impedance spectroscopy (EIS), this measure is useful to predict some electrode properties but does not assess the entire electrode performance while measuring biological signals (Ludwig et al., 2006; Ferguson et al., 2009; Baranauskas et al., 2011). On the other hand, the SNR of the signal recorded through the electrode allows the quantification of the recording performance of the electrode and therefore contrasting electrodes of different types.

Besides electrode limitations, it is important to consider that recording systems have to ensure an amplification stage as close as possible to the recording site by means of a high common mode rejection (the ability to reject common noise in the active and reference electrode) in order to reduce external noise and ensure stable recordings. This can be achieved by a large input impedance in the amplifier (normally in the order of TΩ at 1 kHz) (Nelson et al., 2008). The recorded frequency band is also limited by the filters of the amplifiers, which have to ensure the same amplification value for all the frequencies of interest (Kappenman and Luck, 2010).

Current approaches for assessing SNR in brain recordings rely mostly on the amplitude of the signal. For instance, some reported methodologies are based on the recording of evoked (Kuzum et al., 2014) or spontaneous epileptic activity (Khodagholy et al., 2013) and the SNR is calculated by taking the highest peak during a period of epileptic activity and dividing it by the standard deviation (SD) of the background signal during a period of low biological activity. Recently, a similar procedure was performed by calculating the SNR of slow oscillations—an activity pattern that alternates between periods of neuronal firing, or Up states, and periods of almost neuronal silence, or Down states (Steriade et al., 1993)—as the ratio of the Up state amplitude to the Down state SD (Blaschke et al., 2017). Since these SNR approaches are based on the amplitude of the signal, the calculated SNR values only evaluate the behavior of the devices at the frequency of the recorded events. Nonetheless, obtaining information about the SNR at different frequency ranges of the brain signals is a relevant step in the characterization of recording devices. To overcome the limitations of the current SNR approaches, new methodologies to quantify the SNR in brain recordings are needed.

In brain recordings, the frequency bands of interest include the LFP (<500 Hz), MUA (200–1500 Hz) (Mattia and Del Giudice, 2002) and single-unit activity (>1000 Hz). Within the LFP band are the slow oscillations, which constitute a good model to study the SNR of brain signals because they encompass different frequency bands: from <1 Hz (frequency of Up and Down state alternation) to high-frequency synchronization in the β/γ range (15–100 Hz) (Steriade et al., 1996; Compte et al., 2008) and population spiking activity (MUA) above 200 Hz during Up states (Steriade et al., 1993). Slow oscillations spontaneously arise during slow wave sleep and under anesthesia *in vivo* (Steriade et al., 1993), and also in *in vitro* brain slices (Sanchez-Vives and McCormick, 2000), granting the possibility of exploring them in distinct experimental conditions.

The aims of this study were: (1) to develop and validate a new approach to quantify the SNR of brain recording devices; and (2) to compare the throughput of co-localized electrodes of different materials, namely Au, CNTs and Pt. For these purposes, we used an adaptation of the SNR calculation based on the features of the slow oscillations, which we recorded using MEAs with electrodes of the three aforementioned materials distributed in tritrodes and stereotrodes. The integration of three materials in close vicinity within the same MEA allowed the direct comparison between them, thereby avoiding the problem of comparing electrodes from different probes and/or electronic systems, and avoiding recording different neural activity patterns coming from distant neuronal populations.

## Materials and Methods

### SNR Calculation

Signal-to-noise ratio is defined as the ratio of the power spectral density (PSD) of a signal (meaningful information) with respect to the power of the background noise. In the analysis of brain recordings, this measure is commonly applied in spike sorting to select the best recording location, and also to characterize the reliability of neural information transmission (Schultz, 2007). The SNR can be calculated from spontaneous or evoked neural responses to different types of stimuli (electrical, sensory, etc.). For example, in the calculation of the SNR from a time-dependent signal in single cell recordings, the action potentials are considered “signal” and the inter-spike intervals the “noise.” Then, following the description from Rieke et al. (1997), the SNR is calculated as follows:

(1) $$SNR(f)=\frac{S(f)}{N(f)}$$

Where *S*(*f*) is the PSD of the signal and *N*(*f*) is the PSD of the noise. In our study, we obtained extracellular LFP recordings from active cortical slices that spontaneously generated slow oscillations (Sanchez-Vives and McCormick, 2000). Since Up states are the consequence of a population of neurons firing, we considered Up states the “signal.” As described above, Up states contain a broad band of frequencies; that is, they contain meaningful information. On the other hand, we considered Down states the “noise” because they are mostly silent periods. In order to quantify the SNR at different frequencies, the spectral SNR (in dB) becomes:

(2) $$=10\;\log_{10}\frac{\frac{1}{N}\sum_{i=1}^{N}{\left(PSD_{Up}\right)}_{i}}{\frac{1}{N^{\prime }}\sum_{j=1}^{N^{\prime }}{\left(PSD_{Down}\right)}_{j}}[dB]$$

where *N* is the total number of Up states and *N’* is the total number of Down states.

### SNR Estimators

To easily quantify the performance of each material, thereby avoiding the vast amounts of information obtained through the spectral analysis, we proposed and validated a set of SNR estimators. The advantage of using these SNR estimators is that they reduce and summarize the large amount of information provided by the spectral SNR, since the spectral SNR gives a value of SNR at each different frequency. These estimators are derived from the spectral SNR curve (Eq. 2), or directly from the LFP signal.

#### Area Under the Curve (AUC)

The area under the spectral SNR curve within the frequency range from 5 to 1500 Hz, where 5 Hz is the minimum frequency allowed by the PSD and 1500 Hz is the upper limit of the MUA band. It is calculated as follows:

(3) $$AUC=\int_{fl}^{fu}SNR_{Spectral}(f)df$$

where *fl* is the lower integration limit (5 Hz in this case) and *fu* is the upper integration limit (1500 Hz). However, when AUC is computed in frequency bands, these limits change, being *f*_0_ the lowest frequency of the band and *f* the highest frequency of the band.

The AUC can also be calculated for defined frequency bands. In our case, we chose three frequency bands of interest: low (5–30 Hz), middle (30–200 Hz), and high (200–1500 Hz). The low band ranges from the limit of resolution of the PSD (5 Hz) to the upper limit of the β band (30 Hz). The middle band ranges from the lower limit of the γ band (30 Hz) to the lower limit of the MUA band (200 Hz). Finally, the high band corresponds to a part of the MUA band (>200 Hz).

#### Frequency Limit of Detection (FLOD)

Frequency at which the spectral SNR equals zero. At this point, the power of the signal is exactly the same as the power of the noise.

#### Voltage SNR (vSNR)

This is the most widely reported approach to compute the SNR in LFP recordings in animals under anesthesia (Kuzum et al., 2014; Blaschke et al., 2017). The vSNR is calculated as the ratio between the mean of the peak-to-peak amplitude of all the Up states and the mean of the SD of all the Down states:

(4) $$SNR_{Voltage}=\frac{\frac{1}{N}\sum_{i=1}^{N}{\left(P2P_{Up}\right)}_{i}}{\frac{1}{N^{\prime }}\sum_{j=1}^{N^{\prime }}{\left(STD_{Down}\right)}_{j}}$$

### Fabrication and Characterization of the MEAs

**FIGURE 1:**
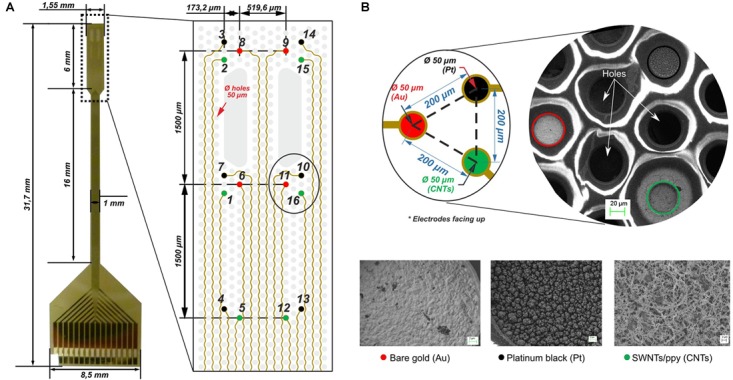
Cortical multielectrode arrays (MEAs). **(A)** Overview of the microfabricated device. Optical microscopy image of the MEA including dimensions and layout in detail of the microelectrodes disposition (tritrodes and stereotrodes) and holes (gray). **(B)** Tritrodes distribution in detail and scanning electron micrographs (SEM) showing 50-μm bare gold microelectrodes before the modification (Au in red), after the modification with platinum black (Pt in black) and the single-walled carbon nanotubes/polypyrrole composite electrode (CNTs in green).

Flexible microprobes integrating 16 Au microelectrodes were fabricated using SU-8 negative photoresist as flexible substrate as previously described (Gabriel et al., 2013; Illa et al., 2015). The fabricated MEA has dimensions of approximately 32 mm long (Figure 1A). The tip, where the array of distributed microelectrodes is, covers 6.00 mm × 1.55 mm. The microelectrodes are distributed in tritrodes and stereotrodes. They are 50 μm in diameter and the center-to-center distance with neighboring electrodes is 200 μm. The rest of the tip is provided with holes to enhance tissue oxygenation. To facilitate the use of the fabricated microprobes, these were connected to a printed circuit board (PCB) with a proper pin output by means of a 16-channel zero insertion force (ZIF) connector. For this, the connecting pads of the microprobe were designed to match the specifications of the desired ZIF connector and, additionally, a spacer was used to ensure good contact between the probe and the connector.

#### Platinum Black (Pt) Electrodeposition

Au electrodes on individual devices were electrochemically coated with a porous layer of platinum black to reduce their impedance through a customized process of platinization (Gabriel et al., 2007). More specifically, electrodeposition was performed using a platinum chloride solution [0.1 M hydrochloric acid, 2.3% platinum (IV) chloride and 0.023% lead (IV) acetate 99%] at -0.2 V for 20 s. The electrodes modified with platinum black correspond to numbers 3, 4, 7, 10, 13, and 14 in Figure 1A, depicted in black. In Figure 1B, a scanning electron microscopy image shows the rough morphology that is achieved by electrodepositing this material.

#### Single-Walled Carbon Nanotubes/Polypyrrole Composite (CNTs) Electrodeposition

Carbon nanotubes were synthesized by the arc-discharge method using graphite electrodes and a Ni/Y 4/1% metal catalyst mixture. As-grown single-walled carbon nanotubes (agSWCNTs; initial nanotube concentration: 4 mg/ml) were dispersed ultrasonically in aqueous 1% sodium dodecylbenzenesulfonates (SDBS) solution. Afterward, the dispersion was centrifuged at 13,000 rpm for 30 min (Hermle Z383, Hermle Labortechnik, Wehingen, Germany) in order to increase their purity and decrease their metal content (Ansón-Casaos et al., 2014), achieving a final nanotube concentration of 1.3 mg/ml.

Electrodeposition of the composite material was carried out in galvanostatic conditions (0.13 mg/ml gSWCNTs, buffer phosphate with 0.05 M dihydrogen phosphate and 0.05 M monohydrogen phosphate solutions, 3.2 mM SDBS and 0.14 M pyrrole) using a constant current value of 3 mA ⋅ cm^-2^ during 120 s. An Ag/AgCl (3 M NaCl) electrode was used as a reference electrode, and a graphite bar was used as a counter electrode.

Figure 1A depicts the electrodes modified with the CNTs composite with the numbers 1, 2, 5, 12, 15, and 16, shown in green. In Figure 1B, a scanning electron microscopy image also shows the rough morphology that is obtained with this coating, and even the tubes can be observed in a random distribution (for further information about the fabrication of the MEAs, see Gabriel et al. (2013).

### *In vitro* Recordings

#### Slice Preparation

This study was carried out in accordance with Spanish regulatory laws (BOE-A-2013-6271), which comply with the European Union guidelines on protection of vertebrates used for experimentation (Directive 2010/63/EU of the European Parliament and the Council of September 22, 2010). The protocol was approved by the ethics committee of Hospital Clinic Barcelona. Two ferrets (5-month-old, male) were anesthetized with sodium pentobarbital and decapitated. The entire forebrain was rapidly removed and placed in oxygenated cold (4–10°C) bathing medium. Coronal slices (0.4-mm thick) from the occipital cortex containing primary and secondary visual cortical areas (areas 17, 18, and 19) were used (Innocenti et al., 2002). A modification of the sucrose-substitution was used to increase tissue viability (Aghajanian and Rasmussen, 1989). Briefly, during the preparation of slices, the tissue was placed in a solution in which NaCl was replaced with sucrose. After the preparation, slices were placed in an interface style recording chamber (Fine Sciences Tools, Foster City, CA, United States). During the first 15 min, cortical slices were superfused with an equal mixture in volume of the normal bathing medium and the sucrose-substituted solution. Next, normal bathing medium was added to the chamber and the slices were superfused for 1–2 h. The modified slice solution was used throughout the rest of the experiment. Bath temperature was maintained at 36°C. The artificial cerebrospinal fluid (ACSF) bathing medium contained (in mM): NaCl, 126; KCl, 2.5; MgSO_4_, 2; NaH_2_PO_4_, 1.25; CaCl_2_, 2; NaHCO_3_, 26; dextrose, 10, and was aerated with 95% O_2_, 5% CO_2_ to a final pH of 7.4. The modified solution had the same ionic composition except for different levels of (in mM): KCl, 4; MgSO_4_, 1 and CaCl_2_, 1–1.2 (Sanchez-Vives and McCormick, 2000). Electrophysiological recordings started after allowing at least 2 h of recovery.

#### Recording Set-Up

Multielectrode arrays attached to a ZIF connector were placed on the slices. The data acquisition system comprised a 16-channel preamplifier (μPA16, Multichannel Systems, Germany) and amplifier (PGA16, Multichannel Systems, Germany) with a 100× gain factor, and a CED 1401 digitizer and Spike 2 software (Cambridge Electronic Design, United Kingdom). The sampling frequency of the recordings was set to 5 kHz.

### Data Analysis

Recordings of 20–60 s duration only from operative tritrodes and stereotrodes were selected for the analysis. From optical imaging and EIS characterization (Figure 1 and Supplementary Figure S1), exclusion criteria were defined: only tritrodes and stereotrodes with the three or two electrodes, respectively, well fabricated and operative were selected for the comparison analysis (some of them were short-circuited or the material was not well deposited). In addition, only recordings with detectable Up states were used. Five MEAs containing four different tritrodes and two different stereotrodes were tested in seven cortical slices. After excluding non-operative tritrodes and stereotrodes (usually a very noisy electrode recording), we ended up with the following sample sizes: *N*_Pt_ = *N*_CNTs_ = 102 and *N*_Au_ = 67 recordings.

Signal analysis was performed using MATLAB 2012a (The MathWorks Inc., Natick, MA, United States). Up and Down state detection was performed as in Castano-Prat et al. (2017). Detection of Up and Down states from the recorded signals was based on three main fingerprints of the Up states: the slow LFP deflection, the gamma rhythm, and the neuronal firing. These three features are reflected in three different time series: (1) the slow oscillation envelope (smoothing filter with a 5-ms moving window), (2) the envelope of the variance of the gamma-filtered LFP (15–100 Hz) (Mukovski et al., 2007), and (3) the estimation of the MUA, which was bandpass filtered from 200 to 1500 Hz (Mattia and Sanchez-Vives, 2012). From each LFP we obtained a highly processed time series as a linear combination of these three features. The contribution of each one was weighted by principal component analysis (PCA). As the three signals correspond to three different frequency bands, this method is very robust against colored noise or band-limited electrode malfunction. Up and Down states were singled out by setting a threshold in this highly processed time series. A threshold calculated from the bimodal distribution of Up and Down states duration was set on the reconstructed signal to classify the parts of the recording with more frequency content (Up states) and less frequency content (Down states). For electrodes where the detection did not work, we used the detection times from the nearest electrode.

Once the detection was performed, the PSD with a resolution of 1024 points of the fast Fourier transform (FFT) was calculated for every Up and Down state separately using Welch’s method (window size 1024 time bins with an overlap of 512 time bins). The mean PSD of the Up states and Down states in the recording fragment were calculated. The same approach was employed to calculate the mean peak-to-peak amplitude of all the Up states and the mean SD of all the Down states.

The Spectral SNR was calculated for every electrode recording using Eq. (2). From the Spectral SNR, AUC was calculated by a trapezoidal numerical integration along the three different defined frequency bands. FLOD was estimated by smoothing the spectral SNR curve to easily find the intersection with zero. The smoothing filter we used is based on a moving average method with a span of 10 ms. vSNR was calculated as the mean peak-to-peak amplitude of Up states divided by the mean SD of the Down states.

For the statistical analyses, the Kolmogorov–Smirnov test was performed for every SNR estimator distribution separately for each material to test the normality. Because none of the distributions was normal, non-parametric tests were applied to assess statistical differences between materials. More specifically, we used the Wilcoxon signed-rank test to compare the SNR distributions of different materials at every frequency, the Mann–Whitney test to assess differences in estimator distributions between different materials, and Pearson correlation coefficient to quantify the degree of association between SNR estimators. A non-parametric ANOVA test equivalent for independent samples (Kruskal–Wallis test) was performed for all the data separately for each MEA, tritrode/stereotrode and material using IBM SPSS 22 statistics software.

## Results

**FIGURE 2:**
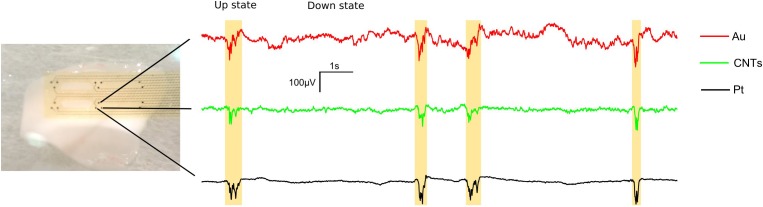
Local field potential (LFP) traces from a representative tritrode showing the recording of spontaneous cortical slow oscillations *in vitro* for the three co-localized electrodes of different materials (Au, CNTs and Pt). For this figure, the LFP signals were filtered by applying Notch filters at 50 and 150 Hz and a smoothing filter using 10-ms sliding windows with 5-ms steps. Up states are shown in yellow.

Five MEAs with electrodes of the three different materials (Au, CNTs and Pt) arranged in tritrodes and stereotrodes were tested on seven different visual cortical slices that generated spontaneous slow oscillations (Figure 2). From the LFP recordings of every electrode, the SNR was calculated using the different methods described above: the spectral SNR, the vSNR estimator, and the estimators derived from the spectral SNR: AUC and FLOD. The proposed SNR analysis was aimed at characterizing the behavior of the three electrode materials while recording brain signals. Moreover, the results themselves validate the proposed SNR analysis as a methodology for characterizing the SNR using biological signals.

### Spectral SNR and AUC Estimator

**FIGURE 3:**
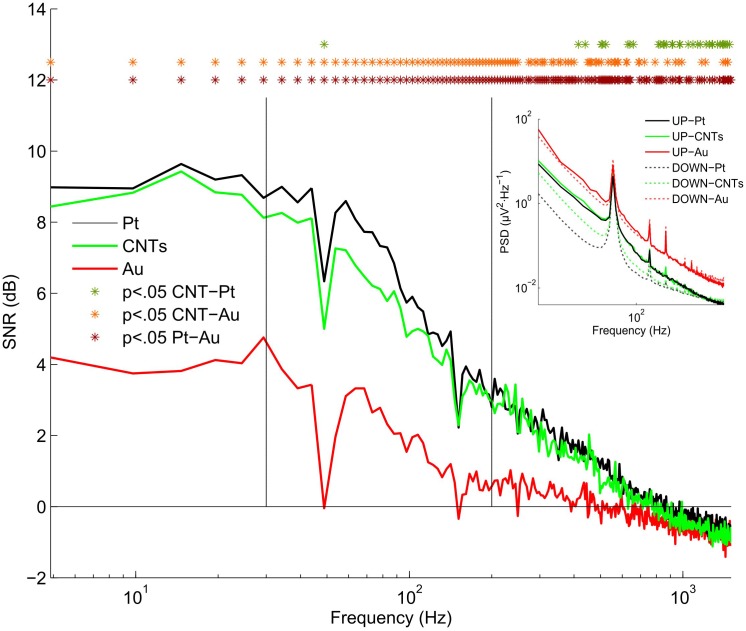
Spectral signal-to-noise ratio (SNR). SNR was calculated as in Eq. (2) giving a SNR value for each frequency ranging from 5 to 1500 Hz. The spectral SNR curve was calculated for the electrodes of the three different materials and the mean SNR curve of the five MEAs was tested in seven slices. The horizontal line at zero represents *SNR* = 0 (*Up*_PSD_ = *Down*_PSD_). Vertical lines separate the three frequency bands: low, 5–30; medium, 30–200; and high, 200–1500 Hz. Inset; Mean power spectral density of Up and Down states (solid and dotted lines, respectively). ^∗^*p* < 0.05 by Wilcoxon signed-rank test.

Since the electrode SNR depends, among other things, on the impedance, the SNR is frequency-dependent. For this reason, measuring the SNR at different biological frequencies is crucial in electrode characterization. The spectral SNR curve was computed for each electrode recording and the results were grouped into materials in order to compare them. Overall, from the spectral SNR analysis, we found that Pt as well as CNTs electrodes showed significantly higher SNR values than Au electrodes for all the functional frequencies (frequencies with SNR > 0 dB) (Figure 3). Moreover, Pt electrodes presented a slightly higher SNR than CNTs electrodes but this difference was not statistically significant for frequencies below 400 Hz. Nevertheless, for certain frequencies above 400 Hz, significant differences appeared between Pt and CNTs electrodes, Pt electrodes having higher SNR values. Regarding the MUA frequency range (200–1500 Hz), Au electrodes showed SNR values very close to zero while Pt and CNTs electrodes had SNR values of approximately 4 dB at 200 Hz (Figure 3). Negative SNR values at higher frequencies are caused because the Down PSD values lightly exceed Up PSD values. This effect may be caused by certain noise artifacts; since Down states have a larger duration than Up states, the probability of having some artifactual noise in Down states is also larger.

More specifically, SNR values for the low-frequency band (5–30 Hz) were almost constant for the three materials (Figure 3). At this frequency range, Pt and CNTs electrodes had SNR values around 9 dB while that of Au was around 4 dB. These values indicate that the power of the signal was around eight times greater than the power of the noise in recordings obtained with Pt and CNTs electrodes and 2.5 times in the case of Au electrodes (*Up*_PSD_/*Down*_PSD_ = 10^SNR/10^). For frequencies over 50 Hz, the SNR decayed almost linearly following the typical 1/*f* decay (Figure 3). The PSD of the Up and Down states (inset in Figure 3) revealed that the power of the Up state was almost the same for Pt and CNTs electrodes while the power of the Down state was lower in Pt electrodes, resulting in better SNR values. The signals recorded by the Au electrodes showed similar powers of Up and Down states, leading to low SNR values.

**FIGURE 4:**
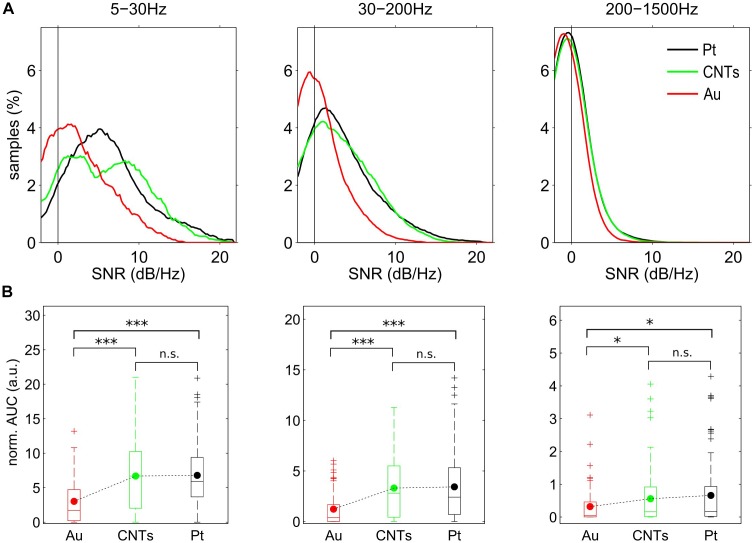
Spectral SNR distribution and area under the spectral SNR curve (AUC) for three different frequency bands. **(A)** Distribution of mean SNR values for the electrodes of the three different materials for the three frequency bands defined in Figure 3. **(B)** Boxplots of AUC in the three frequency bands for the three materials. ^∗^*p* < 0.05, ^∗∗∗^*p* < 0.001 by Mann–Whitney test. *N*_Pt_ = *N*_CNTs_ = 102 and *N*_Au_ = 67 electrode recordings.

The SNR distribution curves represent the mean behavior of different stereotrodes and tritrodes (Figure 4A). For the lower frequency band (5–30 Hz), the distributions were very wide and chi-square shaped while as the frequency increased, the distributions became narrower and closer to SNR = 0, indicating that the recording performance of the electrodes was reduced when the frequency of the signal increased (Figure 4B). In particular, the maximum SNR values in the low frequency band (5–30 Hz) were slightly above 20 dB for Pt and CNTs electrodes, and around 15 dB for Au (Figure 4A, left). While Au SNR distribution peaked at around 2 dB, the peak for Pt was about 5 dB. On the other hand, CNTs electrodes showed a bimodal SNR distribution. The results from the Kruskal–Wallis test (*p* < 0.05) suggest that this bimodality in the SNR value distribution for CNTs electrodes was due to the variability in the fabrication process since significant differences in variances are given by the distribution of SNR values sorted by the tritrode location inside the probe (Supplementary Figure S2). The SNR distribution in the 30–200 Hz frequency range became more similar between Pt and CNTs electrodes, although CNTs distribution was slightly broader (Figure 4A, middle). The distribution for these two materials was wider than that of Au, which was narrow and centered at zero. At the 200–1500 Hz frequency range, the distributions were very similar since most electrodes had their SNR around zero (Figure 4A, right).

The area under the spectral SNR curve (AUC) was calculated as an SNR estimator, and the distributions of normalized AUC values for the three defined frequency bands (low: 5–30 Hz, middle: 30–200 Hz, high: 200–1500 Hz) were represented in boxplots for the three different materials. For the three frequency bands, the AUC for the Pt and CNTs electrodes was significantly higher than for the Au electrodes (*p* < 0.001 for the low and middle frequency bands; *p* < 0.05 for the high frequency band) (Figure 4B). In the 5–30 Hz frequency range, the AUC mean value was equal for Pt and CNTs electrodes but the median was higher for CNTs. On the other hand, for the 200–1500 Hz frequency range, AUC mean values for Pt were greater. At this high-frequency range, the significance values between Pt-Au and CNTs-Au decreased from *p* < 0.001 to *p* < 0.05 because most AUC values were very close to zero, but the difference was still significant.

The results from the spectral SNR analysis shed light on the recording performance of the electrodes for the whole spectral range from 5 to 1500 Hz. The analyzed data show significant differences between Au and Pt, and between Au and CNTs electrodes, indicating that Pt and CNTs electrodes record the brain signals better than Au electrodes for the whole range of studied frequencies. No significant differences were found between Pt and CNTs electrodes but our results suggest that Pt electrodes had a slightly better performance than CNTs electrodes. Finally, our findings validate that the AUC estimator, computed at different selected frequency bands, properly describes the overall behavior of the electrodes in terms of SNR for low, middle, and high frequency ranges.

### vSNR and FLOD as SNR Estimators for Lower and Higher Frequencies, Respectively

**FIGURE 5:**
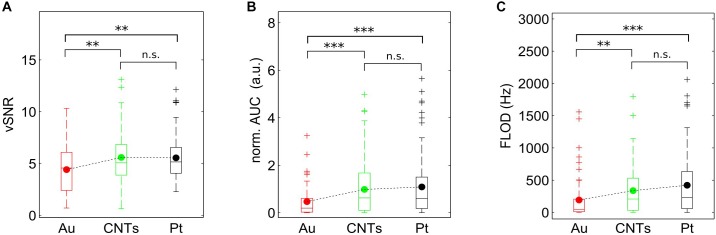
vSNR and FLOD estimators for lower and higher frequencies, respectively. **(A)** Boxplots of the voltage SNR (vSNR) for Au, CNT and PT electrodes. **(B)** Boxplots of the area under the spectral SNR curve (AUC) for the three materials. **(C)** Boxplots of the frequency limit of detection (FLOD) for the three materials. Note the similarities with Figure 4B. ^∗∗^*p* < 0.01, ^∗∗∗^*p* < 0.001 by Mann–Whitney test.

As described above, vSNR and FLOD were calculated as estimators to complement and validate the results obtained with the spectral SNR analysis. Our findings show that vSNR, FLOD, and AUC show the same qualitative results, reinforcing the outcome of the previous SNR analysis (Figure 5).

As vSNR is calculated using the amplitude of the signal during Up states and the SD of the Down states, vSNR values are expected to describe the SNR at frequencies related to the slow oscillations (>1 Hz); that is, very low frequencies. In agreement with this, the results shown in the vSNR boxplot (Figure 5A) match the results obtained with the AUC estimator (Figure 4B) for the low frequency band (5–30 Hz). In addition, the expected tendency of CNTs electrodes to have higher and lower AUC values for lower frequencies and higher frequencies, respectively, than Pt electrodes (Figure 4B) was detected in the vSNR boxplot as well as in the FLOD boxplot (Figures 5A,C).

**FIGURE 6:**
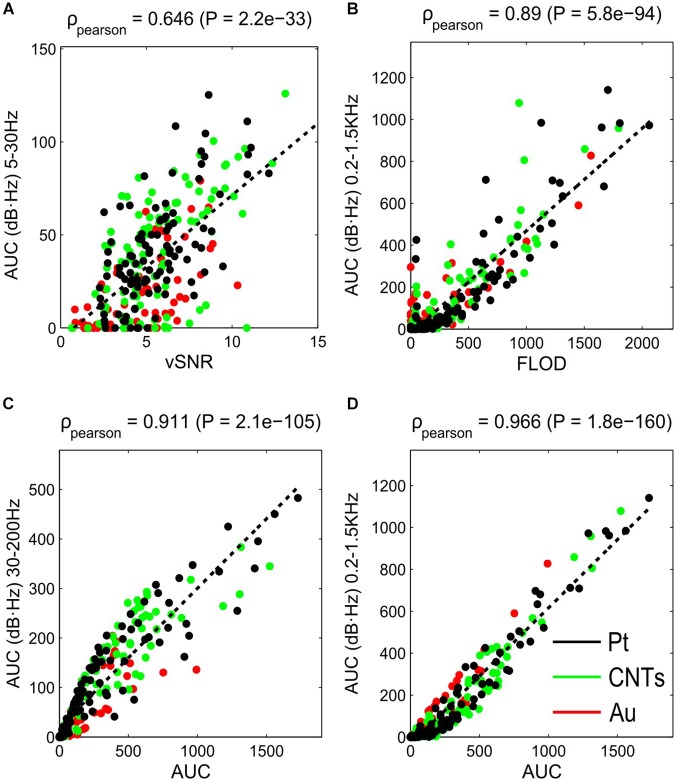
Correlation between SNR estimators (vSNR, FLOD and total AUC) and AUC for different frequency ranges. **(A)** Linear correlation between vSNR and AUC at 5–30 Hz; **(B)** between FLOD and AUC at 200–1500 Hz; **(C,D)** between total AUC and AUC at 30–200 Hz **(C)** and 200–1500 Hz **(D)**. *ρ*, Pearson correlation coefficient.

To confirm that vSNR is related to the SNR at lower frequencies, a linear correlation was performed with the different AUC distributions at the three frequency bands (Figure 6). Since the larger correlation coefficient was for the vSNR-AUC (5–30 Hz), our findings indicate that vSNR better describes the SNR at lower frequencies (Supplementary Figure S3A). Furthermore, vSNR complements our analysis by providing new information regarding the behavior at frequencies that are too low to be obtained with the spectral SNR.

Since FLOD is the value of the frequency at which the SNR is zero, it is easy to relate this estimator to the SNR value at the highest frequencies. Comparing the results of FLOD distributions for each material (Figure 5C) with the distributions of AUC (Figure 4B), one can see that the FLOD estimator has a greater Pearson correlation with AUC at the high frequency range (200–1500 Hz) (Figure 6B) than with AUC at low and middle frequency ranges (Supplementary Figure S3B) Thus, FLOD can be interpreted as an estimator for high frequencies.

The total AUC distribution is dominated by high frequencies (200–1500 Hz) since there are more frequency points within this range and, despite the SNR values being low, the AUC is large due to the frequency variable (note that in the spectral SNR in Figure 3, the frequency axis is in a logarithmic scale). Thus, the total AUC describes the SNR for the middle and high frequencies (Figures 6C,D and Supplementary Figure S3C).

## Discussion

In this work, we have developed and validated a novel method for characterizing the performance of brain recording devices based on a spectral SNR analysis using cortical slow oscillations. The validation was performed by applying this method to characterize and compare electrodes made of three different materials (Au, CNTs and Pt) organized in tritrodes. We also report here that electrodes made of platinum black and carbon nanotubes have better recording performance than electrodes made of gold for the whole functional frequency range that we explored (from 5 to 1500 Hz). Furthermore, Pt electrodes showed a trend toward working better than CNTs electrodes even though the difference did not reach statistical significance. Although these results can be qualitatively predicted from impedance characterization (i.e., directly related to the specific surface area of the electrode site; see Supplementary Figure S1), a quantitative measure of the entire electrode performance can only be assessed by SNR analysis (Ludwig et al., 2006; Baranauskas et al., 2011). We have seen that both functionalized electrodes (CNTs and Pt) displayed lower background noise during the Down states (inset Figure 3); this behavior is due to the reduction of both the root mean square and the thermal noise that are both directly related to the real part of the impedance of the electrode material (Supplementary Table S1). Similar results have already been published describing the improvement in the SNR by using electrodes of electrodeposited platinum black (Desai et al., 2010; Zhang et al., 2013) and carbon nanotubes (Mazzatenta et al., 2007; Keefer et al., 2008; Lu et al., 2010; Baranauskas et al., 2011; Castagnola et al., 2014), reinforcing the validation of our SNR methodology. Nevertheless, a quantitative SNR comparison of the different electrodes recording simultaneously from the same location and using the same recording system has, to our knowledge, not been done before. This novel SNR calculation has also been used recently in the characterization of graphene FET (field effect transistor) arrays in *in vivo* brain recordings, proving the potential of this measure to give information of the recoding capabilities of graphene FET in a broad biological frequency band (Hébert et al., 2017).

The design of the probes was intended to compare the materials while minimizing the interferences derived from recording from different neuronal populations by using co-localized electrodes arranged in tritrodes and stereotrodes. Because our *in vitro* experiments in cortical slices allow the recording of slow oscillations, the intrinsic characteristics of this activity pattern make it suitable for developing a SNR analysis using Up-Down states as Signal-Noise signals, thus overcoming the complexity of the SNR calculation in biological signals that usually arises from the difficulty in separating signal from noise. One could argue that Down states are not totally silent but relatively silent states and therefore that they contain neuronal information (e.g., Reig et al., 2009), such that referring to them as “noise” is not quite accurate. However, notice that in this paradigm there are no experimental manipulations that act on the neuronal firing at any point of the experiment, plus the recordings from closely located electrodes of different materials are compared. Under these conditions, it seems reasonable to assume that Down states be considered “noise” with respect to Up states. Thus, the probes design, as well as the experimental approach, allowed the development of this SNR analysis to be able to compare quantitatively the behavior of electrodes made of different materials.

There are some differences between measurements carried out by injecting artificial currents in saline solution electrolyte (as in EIS; see Supplementary Figure S1) in contrast to *in vivo* and *in vitro* measurements. Measurements in saline solution display lower impedance values and larger capacitive charge capacity in comparison with the measures where the electrodes are in contact with tissue (Wei and Grill, 2009). This effect can be attributed to the differences in the composition between both electrolytes (saline solutions and brain tissue) and the difference in the electrode-reference path. When using saline solution as an electrolyte both electrode and reference are soaked in the same electrolyte. In an *in vitro* preparation, the electrode is placed over the slice of brain tissue and the reference is placed in the ACSF bath. In this case the path is composed of the brain slice and the ACSF bath. Finally, in an *in vivo* preparation, the electrode contacts the brain and the reference can be placed in different locations, such as the nearby muscles. In this case, the path is composed of the brain, the skull and the muscle. Normally, as the electrode-reference path increases, so does the number of different electrolytes (with different electrical properties), and the noise of the measured signal increases. In conclusion, both electrode–electrolyte interaction and electrode–reference path, determine the impedance-frequency dependence and the level of noise (both thermal and RMS – root mean square).

The results from the spectral SNR analysis provide a large amount of data since they give an SNR value for each frequency. Therefore, the use of SNR estimators that give an overall idea of the behavior under certain conditions is especially helpful. Because of this, we defined and validated some SNR estimators extracted from the spectral SNR analysis. On the one hand, we defined several SNR estimators from the spectral SNR analysis: total AUC, the AUC for different frequency bands of biological interest (5–30 Hz, 30–200 Hz and 200–1500 Hz), and the FLOD. On the other hand, we also calculated the vSNR, which is the standard approach to calculating SNR in LFP recordings (Khodagholy et al., 2013; Kuzum et al., 2014; Blaschke et al., 2017). AUC values defined in frequency bands of interest are very useful estimators for describing and quantifying the behavior of the electrodes (Figure 3). FLOD and vSNR estimators were compared with the AUC at different frequency bands and we found that while FLOD describes very well the SNR at high frequencies, the vSNR describes the SNR better at lower frequencies. Proving our hypothesis that vSNR applied to these slow oscillation signals describes the SNR at the slow oscillations frequency (<1 Hz), the correlation of this estimator with AUC at different frequencies is highest for the lower frequencies. On the other hand, vSNR can give more information of the SNR values at lower frequencies where spectral SNR cannot reach enough frequency resolution due to the short duration of Up states (∼200 ms). Furthermore, we observed that the total AUC describes the SNR behavior at middle-high frequencies. This can be explained because, given the nature of the spectral analysis, the AUC is more weighted toward higher frequencies. Since we defined lower frequencies as 5–30 Hz, only six frequency values were considered, while for the 200–1500 Hz range, 266 frequency values were considered. This is why we normalized the AUC in different frequency bands by the number of frequency values.

Searching in the literature for SNR calculation methods, we found two different methodologies estimating the SNR at different frequencies that we consider to be worthy of note. The first one involved estimating the SNR from recordings of spike and wave discharges (SWDs) during spontaneous epileptiform activity in an animal model of absence epilepsy (Khodagholy et al., 2013). The authors of this study calculated the SNR as the ratio between the power spectra of the recording during ictal seizure in SWDs and the power spectra of the recording during inter-ictal activity (period of low biological activity) (Khodagholy et al., 2013). The second methodology implied the calculation of the SNR as the ratio between the power of the signal from the recording of rapid-eye movement (REM) sleep and the power of the signal from post-mortem recordings (Khodagholy et al., 2015). The main drawback of the first approach is the need for a specific transgenic animal model in order to have a signal of large enough amplitude, which will nevertheless be a pathological one. The possible changes that arise due to obtaining the ictal and the inter-ictal activity recordings at different time periods and the variability of SWDs activity across different animal subjects and levels of anesthesia makes this approach less robust. One of the drawbacks of the second approach is that REM activity is very similar to activity during wakefulness and the only way to distinguish one from the other is by means of an electromyogram (EMG) recording. Furthermore, in contrast to the recording of a live animal, in a post-mortem recording there is no respiration or heartbeat; finally, other biological changes related to the cessation of the homeostatic equilibrium occur. Therefore, these changes could lead to a bias in the SNR calculation.

In order to overcome these drawbacks, we propose a SNR methodology based on the slow oscillatory state that arises during anesthesia and during non-REM sleep in typical *in vivo* conditions and can be also reproduced in the *in vitro* preparations. The SNR can be assessed at different frequencies since we are applying a spectral analysis, considering the signal in the Up states (given that they contain different biological frequency rhythms caused by the firing of populations of neurons), and the noise in the Down states (since they are periods of almost silent neuronal firing). The alternation between these two states in short time periods make the approach more robust than the two approaches described above. In addition, the spectral SNR analysis was performed using the mean PSD from different Up and Down states occurring in less than 1 min, conferring higher statistical reliability than the other methods that only used one large period as signal and one large period as noise, with both periods being very separated in time from each other. In other words, the proposed SNR methodology has the advantage of allowing the quantification of the SNR for different frequencies by using the Up and Down states of the well-known slow oscillations, which are the default neural activity pattern during sleep and anesthesia and are reproducible in *in vitro* preparations. To sum up, we have developed a novel and robust method to quantify the performance of electrodes in brain recordings by a novel SNR approach adapted to cortical slow oscillations. Additionally, we have validated the approach by quantifying the performance of electrodes made of three different materials by recording electrophysiological signals from the brain, showing that platinum black as well as carbon nanotubes electrodes have better working performance than gold electrodes.

To sum up, we present a detailed SNR analysis to quantify and compare the performance of different devices to record brain activity. Neural MEAs with electrodes of different materials arranged in co-localized tritrodes and stereotrodes were used to record slow oscillations from the cerebral cortex network. This approach was designed to avoid the interferences from external variables and thus enable a proper comparison between electrodes. The results shed light on the recording behavior of electrodes made of different materials in a broad range of biological frequencies showing that platinum black as well as carbon nanotubes electrodes have better working performance than gold electrodes. Furthermore, the results obtained here parallel previous studies involving some of the tested materials, hence reinforcing the validation of the proposed SNR approach. The work here exposed is also intended to validate and standardize a methodology for quantifying the SNR in different types of brain recording devices such as electrodes or transistors.

## Author Contributions

GG and MVSV designed the MEA and tritrodes. GG, XI, AGB, JHF, MM, and RV fabricated and characterized the MEAs. BR and MVSV designed and performed the *in vitro* experiments. AS-P developed the SNR measure and performed the data analysis. All authors wrote the paper.

## Conflict of Interest Statement

The authors declare that the research was conducted in the absence of any commercial or financial relationships that could be construed as a potential conflict of interest.
