# Supplementary material for: Quantification of Signal-to-Noise Ratio in Cerebral Cortex Recordings Using Flexible MEAs With Co-localized Platinum Black, Carbon Nanotubes, and Gold Electrodes
Source: Front Neurosci. 2018 Nov 29;12:862. doi: 10.3389/fnins.2018.00862 (PMC6282047; doi:10.3389/fnins.2018.00862)
Supplement: Supplementary file 1 [file Data_Sheet_1.PDF]

Supplementary Material

Quantification of signal-to-noise ratio in cerebral cortex recordings using flexible MEAs with co-localized platinum black, carbon nanotubes and gold electrodes

Alex Suarez-Perez, Gemma Gabriel, Beatriz Rebollo, Xavi Illa, Anton Guimerà-Brunet, Javier Hernández-Ferrer, Mª Teresa Martínez, Rosa Villa, Maria V. Sanchez-Vives*

***Correspondence**:
E-mail: msanche3@clinic.ub.es

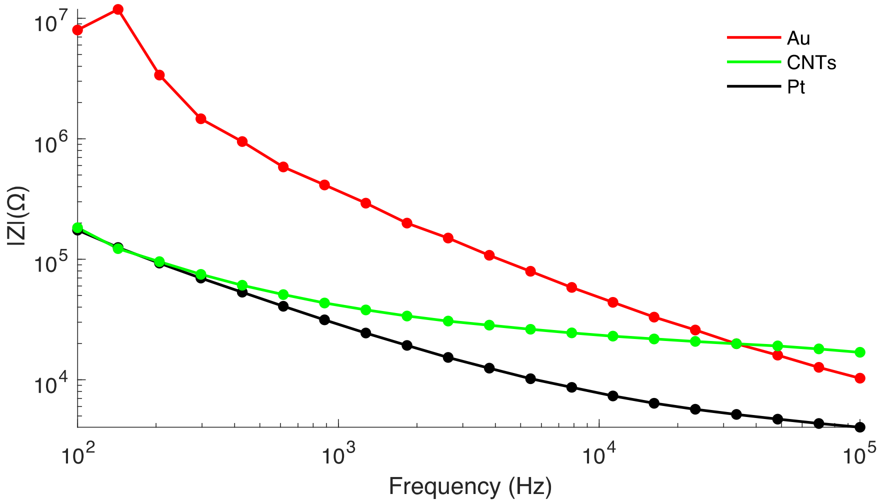

Figure S1; Electrochemical impedance spectroscopy (EIS) characterization of the cortical multielectrode arrays (MEAs). Magnitude of the impedance (|Z|, in Ohms) measured at different frequencies for the electrodes made of the three different materials: Au, CNTs and Pt.

Parameter	Unit	Au	CNTs	Pt
|Z| (at 1kHz)	kΩ	396.2 ± 287.0	63.9 ± 11.2	39.3 ± 64.8
Rs	kΩ	10.3 ± 3.5	19.3 ± 12.6	3.76 ± 2.2
Thermal Noise (ca 10^5 Hz)	µV	4.1 ± 2.4	5.6 ± 4.6	2.5 ± 1.9
RMS Noise (100-1500 Hz)	µV	7.1 ± 7.8	1.8 ± 2.8	1.3 ± 1.8

Table S1. ExExperimental results from electrochemical impedance spectroscopy characterization showed in figure S1. Magnitude of the impedance (|Z|) at 1 kHz; Rs as the resistance at phase angle zero (value of the impedance at 10^5 Hz); Thermal Noise defined by $V_{noise} = \sqrt{4 \cdot K_B \cdot T \cdot R \cdot \Delta f}$, where K_B is the Boltzmann constant, T is the temperature in Kelvin, R is the resistance or the real part of the electrode impedance (Rs), and Δf is the recorded frequency range. RMS noise is defined by $V_{rms} = \sqrt{4 \cdot K_B \cdot T \cdot \int_{f_0}^{f_1} |Z(f)| df}$ for reactive impedances (Nyquist 1928).

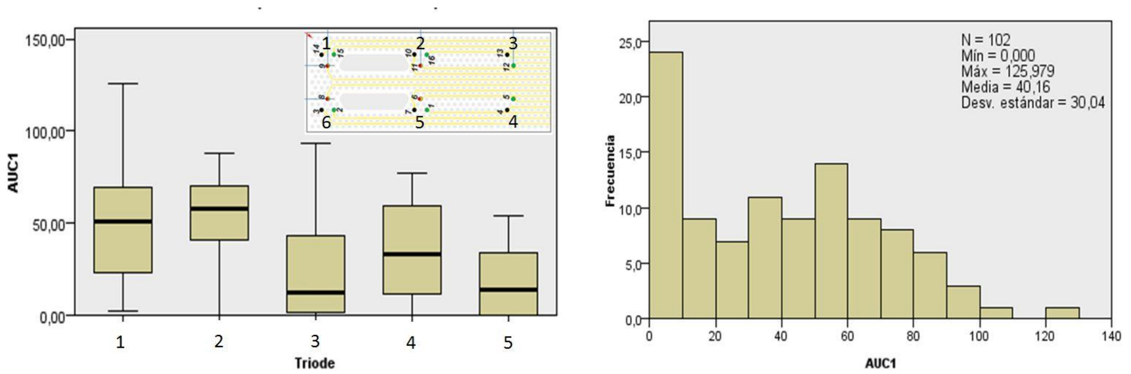

Figure S2; **Bimodal distribution of SNR values for CNTs electrodes at the low frequency range (5-30 Hz) caused by differences in the tritrode location in the probe.** Kruskal-Wallis test applied for AUC (from 5 to 30 Hz) shows significant differences between tritrodes (*p*<0.05): **(A)** Boxplot of the AUC of the low frequency range (5-30 Hz) for the different tritrodes and stereotrodes in the probe showing two homogeneous subsets (subset 1: tritrodes 1, 2 and stereotrode 4; subset 2: stereotrode 3 and tritrode 5). **(B)** Histogram of AUC values at the low frequency band (5-30 Hz) for CNTs electrodes showing the bimodal distribution.

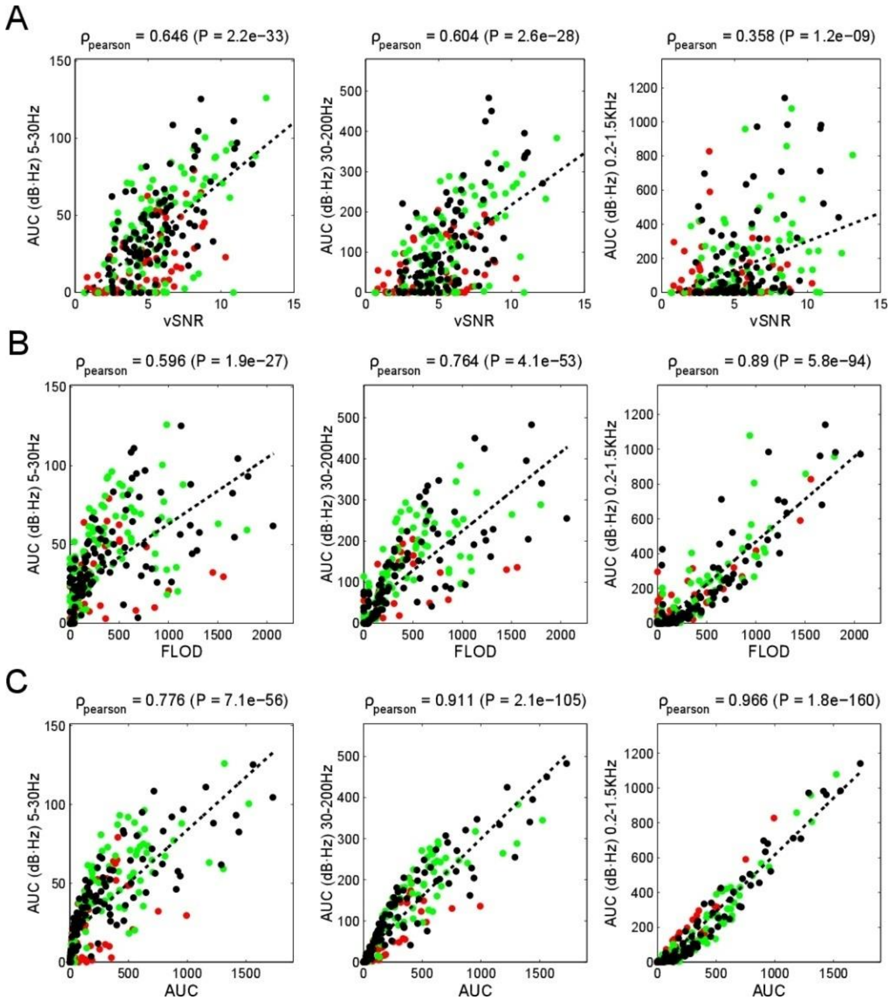

Figure S3; **vSNR, AUC and FLOD as SNR estimators of low, middle-high and high frequency signals, respectively.** Linear correlation between: **A)** vSNR and AUC, **B)** FLOD and AUC, and **C)** total AUC and AUC at three defined frequency ranges: 5-30 Hz (left), 30-200 Hz (middle) and 200-1500 Hz (right). Pearson correlation coefficient (ρ) calculated to estimate the degree of correlation between variables.

<u>Bibliography</u>

Nyquist, H. (1928). Thermal agitation of electric charge in conductors. *Physical review*, *32*(1), 110.
